# The Nasogastric Tube for Adhesional Small Bowel Obstruction: An Analysis of Treatment Effect and Outcomes in a Tertiary Acute General Surgical Unit

**DOI:** 10.7759/cureus.76163

**Published:** 2024-12-21

**Authors:** Ali Al-Mashat, Mostafa Elkhawaga, Stephen Smith, Jonathan Gani, Christine O'Neill, David Burnett, Rosemary Carroll, Natalie Lott, Peter Howley, Humaira Mahin

**Affiliations:** 1 General Surgery, John Hunter Hospital, Newcastle, AUS; 2 Hunter Surgical Clinical Research Unit, John Hunter Hospital, Newcastle, AUS; 3 General Surgery, Calvary Mater Hospital, Newcastle, AUS; 4 School of Medicine and Public Health, University of Newcastle, Newcastle, AUS; 5 Faculty of Medicine, Health and Human Sciences, Macquarie University, Sydney, AUS

**Keywords:** adhesions, conservative vs. surgical management, nasogastric tube, retrospective research, small bowel obstruction

## Abstract

Background

Nasogastric tubes (NGTs) have long been considered standard practice for the management of adhesional small bowel obstructions (ASBOs). However, the evidence to support the routine use of NGTs in ASBO is sparse. This study aims to review outcomes associated with NGT use in a large tertiary centre.

Methods

Electronic medical records from patients diagnosed with an ASBO admitted to the acute general surgical unit at John Hunter Hospital between January 2016 and December 2020 were reviewed. Data were analysed to compare outcomes between groups receiving an NGT and those not receiving an NGT.

Results

A total of 460 admissions based on 387 patients with ASBO were analysed. An NGT was used in 264 (57%) admission episodes. Higher American Society of Anaesthesiologist scores were seen in the NGT group (p=0.0265). Surgical intervention was required in 94 (36%) patients who had an NGT inserted compared to 13 (7%) patients without an NGT (p<0.0001). The average length of stay was 6.9 days (95% confidence interval [CI], 6.1-7.6) in the NGT group versus 3.2 days (95% CI, 2.7-3.7) in the no-NGT group (p<0.0001). The mean time to surgery was 58.8 hours (95% CI, 47.4-70.3) in the NGT group versus 19.4 hours (95% CI, 12.2-26.5) in the no-NGT group (p=0.0163). Patients in the NGT group were more likely to undergo bowel resection (8% versus 2%; p=0.023).

Conclusions

This five-year retrospective study demonstrates that patients with an NGT for ASBO exhibited poorer outcomes than those without one. While these results may reflect a selection bias towards patients with greater disease severity, they highlight the need for further evaluation through a randomised controlled trial.

## Introduction

The use of nasogastric tubes (NGTs) to treat adhesional small bowel obstruction (ASBO) by decompressing the stomach dates back to the 1920s. Abraham Levin is credited with inventing the NGT in 1921 [1]. Owen Wangensteen first described using the NGT routinely for ASBO in the 1920s [2]. He later published his results in the 1930s, indicating that the non-operative management of ASBO with NGTs was associated with lower mortality than with routine surgery [3]. Wangensteen’s landmark description and publication resulted in him being awarded the prestigious Samuel Gross prize and in NGT decompression becoming a standard of care for the management of ASBO [4,5]. Since this description in the 1930s, there has not, to our knowledge, been a prospective clinical trial comparing the use of NGTs against a control group managed without an NGT in the non-operative management of patients with ASBO. While two randomised controlled trials (RCTs) in the literature compare NGT use with longer triple-lumen intestinal tubes [6,7], with differing results, no RCTs exist comparing NGT to no-NGT intervention.

While no RCTs exist on the topic, there are retrospective studies suggesting that the use of NGTs may be associated with higher rates of pneumonia, respiratory failure, and operative intervention [8-10]. Furthermore, there is Level 1 evidence that NGTs are associated with poorer outcomes following elective gastrointestinal surgery [11]. The combination of these factors and the fact that there remains significant risk associated with the use and placement of NGTs [12] suggest that the routine use of NGT in ASBO should be examined. This paper documents a retrospective review of all patients presenting to a tertiary referral acute general surgical unit (AGSU), with ASBO over a five-year period, comparing outcomes between those who had NGT insertion and those who did not.

## Materials and methods

Clinical setting

The AGSU at the John Hunter Hospital (JHH), Newcastle, was set up in 2008. JHH serves as the designated tertiary referral facility for the Hunter New England Local Health District, encompassing a region spanning 131,785 square kilometres and a population of approximately 920,370 residents. All patients with ASBO (either presenting to the ED or being transferred from smaller centres for tertiary referral centre care) are admitted under the JHH AGSU. The AGSU surgical consultant admitting roster provides a 24-hour, 7-day a week cover, with handover at 7 pm, a 12-hour on-site consultant-led service (7 am to 7 pm), and an afternoon operating theatre list (1 pm to 5 pm). Patients with ASBO are protocolised to remain under the AGSU for 48 hours. If a decision is made to operate at any stage, the patient is taken off the AGSU and placed under the operating surgeon’s bedcard. If the decision is made to continue with non-operative management after 48 hours, the patient is transferred to the bedcard of the surgeon for AGSU at that time. Water-soluble contrast was routinely administered the morning after admission for all patients with ASBO. NGT placement was not routine, and the decision to place an NGT was made by any member of the AGSU team or the ED team.

Study design

A five-year retrospective analysis was conducted on data from January 2016 to December 2020, involving the review of electronic medical records through the clinical admission portal database. Patients aged 18 or above with ASBO admitted at JHH and a history of previous abdominal surgery were included. The diagnosis of ASBO was established either through clinical evaluation (abdominal pain, distention, obstipation, and vomiting) or imaging (abdominal X-rays and/or CT scans). Patients were excluded if they received prompt surgical intervention for conditions such as bowel ischemia, peritonitis, or strangulation, had other identifiable causes of small obstruction such as hernias, malignancies, foreign bodies, and inflammatory strictures, were receiving palliative care, or were diagnosed with ASBO while already hospitalised for a different primary reason.

Outcome measures

Comparison was made between patients who had an NGT inserted during their admission for ASBO and those who did not. Those patients who had an NGT placed only after the decision was made to operate (identified through examination of operative records) were kept in the ‘no-NGT’ group for the purposes of analysis. The predetermined outcomes assessed were: the need for surgical intervention, the rate of bowel resection, the time to surgery from admission, the length of hospital stay (LOS), the rate of NGT-related complications and the mortality rate.

Statistics

Patient characteristics were summarised using descriptive statistics by NGT status. Comparisons of means were assessed using Welch’s Analysis of Variance to allow for unequal variances. Tests for associations were assessed via Chi-squared or Fisher’s exact tests. P-values of less than 0.05 were considered statistically significant.

## Results

Around 460 admissions with ASBO (based on 387 individual patients) were included in the study, of which 422 (92%) were admissions directly to JHH, with the remainder (38) via transfer from another hospital. Of the total admissions, 264 (57%) involved the use of an NGT for ASBO.

Approximately 46% of admissions were male. The average age of patients receiving an NGT was 63.5 years (median 68) compared to 59.4 years (median 64) in the no-NGT group. Approximately 52% of males and 62% of females had an NGT inserted. Higher American Society of Anaesthesiologists (ASA) scores were associated with NGT insertion (p=0.0265, Table 1).

**Table 1:** Comparative analysis of patient demographics and characteristics in NGT vs. no-NGT groups ASA, American Society of Anaesthesiologists; ASBO, Adhesional small bowel obstruction; JHH, John Hunter Hospital; NGT, Nasogastric tube

Patient demographics and characteristics		NGT (n=264 [57%])	No-NGT (n=196 [43%])	p-value
Age (mean)		63.5 years	59.4 years	0.0157
Male (n, %)		110 (52%)	101 (48%)	0.0358
ASA score (n, %)	1	18 (7%)	10 (5%)	0.0265
	2	106 (40%)	100 (51%)	
	3	93 (35%)	70 (36%)	
	4	47 (18%)	16 (8%)	
Direct admission to JHH		232	190	0.0002
1^st^ presentation with ASBO (n, %)	Yes	151 (57%)	94 (48%)	
	No	86 (33%)	93 (47%)	0.0080
	Unknown	27 (10%)	9 (5%)	
Imaging conducted		262	193	0.0777

**Figure 1:**
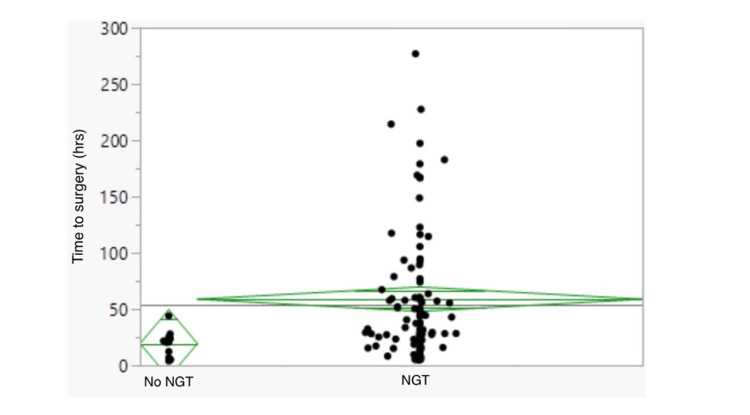
Time to surgery from admission time as per NGT status (hours) The horizontal line across the middle of the green diamond represents the mean; the top and bottom of the green diamond indicate the 95% confidence interval for the mean. NGT, Nasogastric tube

A total of 107 patients were identified as requiring an operation for ASBO during their admission. Surgical intervention was associated with NGT placement, with 94 out of 264 (36%) patients in the NGT group having an operation compared to 13 out of 196 (7%) patients who did not have an NGT (p<0.0001, Table 2). This association persisted with analysis of those patients admitted directly to JHH only. The bowel resection rate was higher in the NGT group (21/264 [8%] versus 3/196 [2%], p=0.023, Table 2). Excluding one outlier of 456 hours in the no-NGT group, the mean time to surgical intervention was longer in the NGT group (58.8 hours [95% confidence interval {CI}, 47.4-70.3] versus 19.4 hours [95% CI, 12.2-26.5], p=0.0163, Table 2, Figure 1).

**Table 2:** Outcome comparison between NGT and no-NGT groups *Excluding one outlier of 456 hours in the no-NGT group CI, Confidence interval; NGT, Nasogastric tube; SD, Standard deviation; SEM, Standard error of the mean

Outcome		NGT (n=264 [57%])	No-NGT (n=196 [43%])	p-value
Surgical intervention (n, %)		94 (36%)	13 (7%)	<0.0001
Non-operative (n, %)		170 (64%)	183 (93%)	<0.0001
Bowel resection (n, %)		21 (8%)	3 (2%)	0.0230
Length of hospital stay (days)	Mean +/- SEM	6.9 +/- 0.39	3.2 +/- 0.28	<0.0001
	SD	6.4	3.9	
	95% CI	6.1-7.6	2.7-3.7	
Time to surgery (hours)*	Mean +/- SEM	58.8 +/- 5.76	19.4 +/- 3.25	0.0163
	SD	55.6	11.3	
	95% CI	47.4-70.3	12.2-26.5	
Mortality (n, %)		3 (1%)	2 (1%)	1.0000

**Figure 2:**
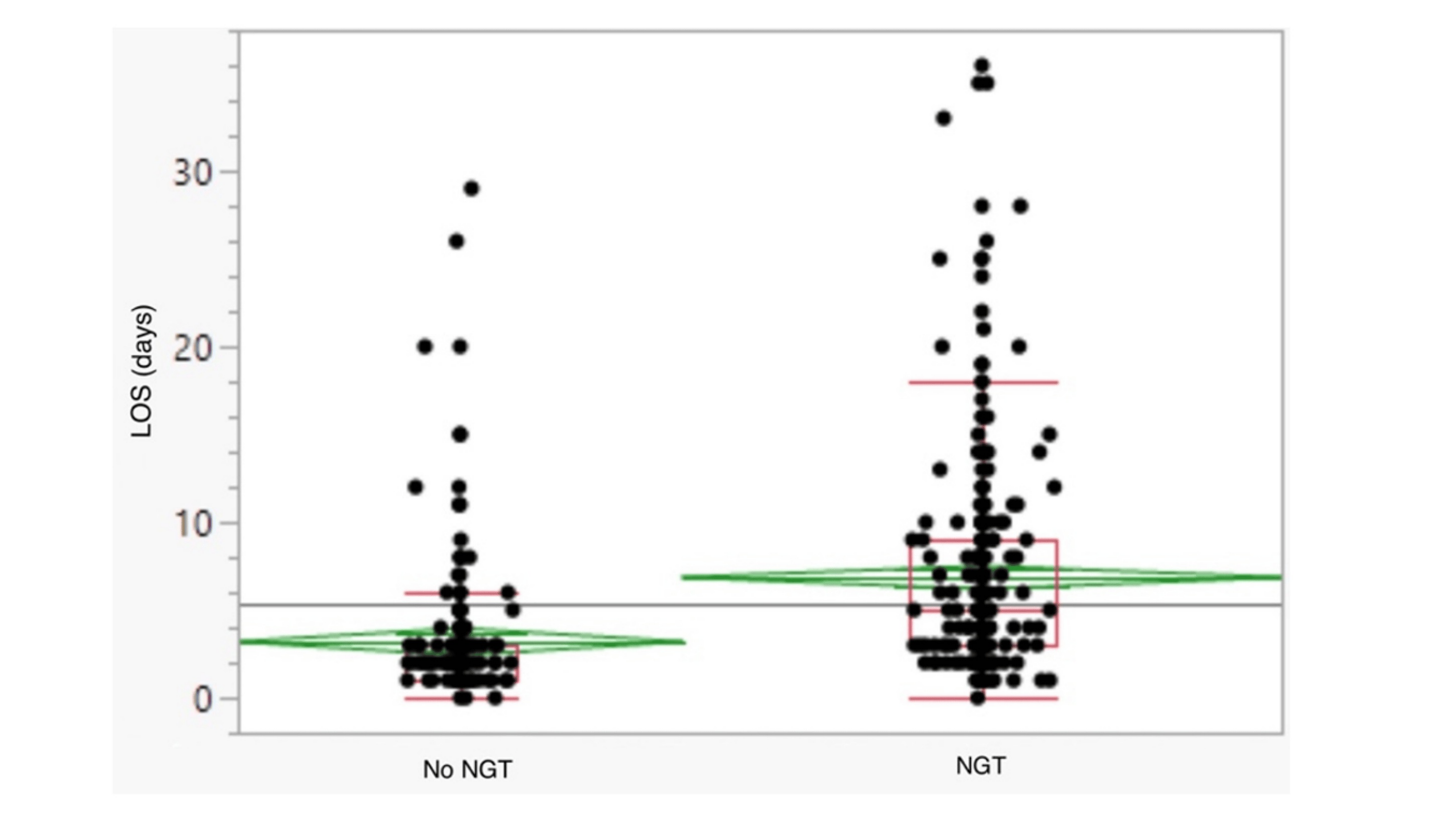
LOS as per NGT status (days) Horizontal line across the middle of the green diamond represents the mean; the top and bottom of the green diamond indicate the 95% confidence interval for the mean. Red horizontal lines indicate 5th, 25th, 50th, 75th, 95th percentiles. LOS, Length of hospital stay; NGT, Nasogastric tube

The overall LOS was longer in the NGT group with a mean of 6.9 days (95% CI, 6.1-7.6) compared with 3.2 days (95% CI, 2.7-3.7) in the no-NGT group (p<0.0001, Figure 2, Table 2). In the NGT group, 27 (10%) admissions involved rehabilitation support compared with 5 (3%) admissions where an NGT was not used (p=0.0019). Two of the 196 no-NGT (1%) admissions and three of the 264 (1%) NGT-related admissions resulted in a death (p=1.0000).

NGT-related complications were recorded in 18% of patients receiving an NGT with the most common being pulmonary related (74%). Seventeen patients developed an aspiration pneumonia after NGT insertion while there were 17 recorded cases of atelectasis. Additional complications included oesophagitis (n=2), gastric reflux (n=1), sinusitis (n=2), pharyngitis (n=6), tube knotting (n=1), incorrect tube positioning (n=1), and laryngitis (n=1).

## Discussion

This retrospective study demonstrates that patients admitted with ASBO who underwent NGT placement as part of their management exhibited a higher likelihood of requiring surgery, a greater likelihood of undergoing bowel resection, experienced a longer time to undergo their operation, and had an extended hospital stay. This study did not use score matching or propensity score analysis; therefore, the results may include selection bias. The higher ASA scores observed in the NGT group suggest that the findings may largely reflect a clinician selection phenomenon (i.e., sicker patients being more likely to have an NGT inserted). However, a direct relationship between NGT insertion and poorer outcomes is also possible and cannot be excluded without a prospective clinical trial.

Despite being universally accepted and part of the Bologna guidelines on the management of ASBO that decompression with NGT is standard practice [5], our review of the literature appears to show that there are no RCTs or prospective cohort studies comparing NGT to no-NGT for the management of ASBO. There are, however, three retrospective studies that have shown poorer outcomes in NGT patients. Fonseca et al. analysed 290 patients with ASBO and found an increased risk of pneumonia, respiratory failure, and LOS associated with NGT use [8]. Berman et al. studied a group of 181 patients with ASBO and found that NGT usage resulted in no difference in rates of surgery or the need for a bowel resection but did result in a two-day increased LOS [9]. Shinohara et al investigated 288 patients, finding no significant differences in risk of pneumonia, need for surgery, or rates of vomiting after admission, even among patients with larger gastric volumes, but noted prolonged hospital stays and delayed time to oral intake in the NGT group [10].

When evaluating the establishment of NGT as the standard of care for managing ASBO, there are two key factors to consider. First, the rationale behind decompressing gas and liquid contents to relieve symptoms and potentially reverse the obstruction appears logical. The second is the dramatic mortality results seen with the introduction of the NGT for ASBO. Wangensteen’s initial research showed that mortality rates from ASBO dropped from 50% to 18% after the NGT was introduced. On closer examination of these results, which were seen at the University of Minnesota over many years, there may be some other factors that were also responsible such as careful attention to fluid and electrolytes, the realisation that it was intestinal ischemia rather than a toxin that caused most of the mortality, the introduction of antibiotics and the fact that patients were treated conservatively instead of universally operated on [2-4,13].

Almost half the patients in this study had no NGT (196 versus 264) inserted for the purpose of ASBO. This represented a lower rate of insertion than the authors expected but highlights the fact that the routine use of NGT for ASBO is evolving, with surgeons already adopting the selective use of nasogastric intubation. This is probably the most likely explanation for the association of higher rates of operative intervention in the NGT group. However, it is still possible, based on these results, that NGT insertion is at the very least failing to fulfil its anticipated role as a therapeutic intervention, given there were higher rates of bowel resection, a longer time to undergo their operation, and an extended hospital stay associated with its use.

The results from our study seem to be at odds with the widely held view that NGT decompression is beneficial for the ASBO patient. These results may not be quite as controversial as they seem. Only 20-30 years ago, routine NGT decompression was considered standard care following elective small and large intestinal resectional surgery. It is no longer used for this indication due to research highlighting increased respiratory complications, a slower return of bowel function, and a longer LOS associated with its use post-operatively [11]. It is logical and proven physiologically that NGT tubes increase respiratory complications by causing both regurgitation and aspiration. Regurgitation episodes occur as a result of the tube, keeping the lower sphincter mechanism open and increasing the frequency of its relaxations, while aspiration occurs from the upper oesophageal sphincter being kept open by the tube and desensitisation of the pharyngo-glottic adduction reflex [14,15]. Bowel function is a complex mechanism, but certainly, one of the main triggers for chemical and neurological stimulation of the more distal gastrointestinal tract is a full stomach [16]. It stands to reason that emptying the stomach with an NGT could slow down recovery after surgery in the acute setting and following elective surgery.

There are several limitations to this study. Like all retrospective surgical studies, it is very difficult to factor in the decision-making process when assessing the outcomes alone. It is also unlikely that all ASBOs are created equal; the clinician might recognise some aspect of the presentation that fulfils their own criteria for NGT insertion. This cannot be accurately surmised by a retrospective review. However, this study represents the largest study on the topic, with the highest rate of patients treated without an NGT. Considering the unfavourable outcomes associated with NGT use, along with the absence of substantial supporting evidence, the existing paradigm of universal NGT use must be called into question. An RCT on this topic is long overdue.

## Conclusions

This five-year retrospective study demonstrates that patients with greater disease severity were more likely to receive an NGT, and those who received an NGT experienced poorer outcomes compared to those who did not. While this may reflect a clinician selection phenomenon, a more direct relationship with NGT use is also possible. Further evidence in the form of an RCT is needed.
